# Magnesium homeostasis in colon carcinoma LoVo cells sensitive or resistant to doxorubicin

**DOI:** 10.1038/srep16538

**Published:** 2015-11-13

**Authors:** Sara Castiglioni, Alessandra Cazzaniga, Valentina Trapani, Concettina Cappadone, Giovanna Farruggia, Lucia Merolle, Federica I. Wolf, Stefano Iotti, Jeanette A M Maier

**Affiliations:** 1 Dipartimento di Scienze Biomediche e Cliniche L. Sacco, Università di Milano, Via G.B. Grassi 74, Milano I-20157; 2 Istituto di Patologia Generale, Facoltà di Medicina, Università Cattolica del Sacro Cuore, Largo F. Vito 1, Roma I-00168; 3 Dipartimento di Farmacia e Biotecnologie, Università Alma Mater di Bologna, Via San Donato 19/2, Bologna I-40127; 4 Istituto Nazionale Biostrutture e Biosistemi, Viale delle Medaglie d’oro 305, Roma I-00136

## Abstract

Neoplastic cells accumulate magnesium, an event which provides selective advantages and is frequently associated with TRPM7overexpression. Little is known about magnesium homeostasis in drug-resistant cancer cells. Therefore, we used the colon cancer LoVo cell model and compared doxorubicin-resistant to sensitive cells. In resistant cells the concentration of total magnesium is higher while its influx capacity is lower than in sensitive cells. Accordingly, resistant cells express lower amounts of the TRPM6 and 7, both involved in magnesium transport. While decreased TRPM6 levels are due to transcriptional regulation, post-transcriptional events are involved in reducing the amounts of TRPM7. Indeed, the calpain inhibitor calpeptin markedly increases the levels of TRPM7 in resistant cells. In doxorubicin-sensitive cells, silencing *TRPM7* shifts the phenotype to one more similar to resistant cells, since in these cells silencing *TRPM7* significantly decreases the influx of magnesium, increases its intracellular concentration and increases resistance to doxorubicin. On the other hand, calpain inhibition upregulates TRPM7, decreases intracellular magnesium and enhances the sensitivity to doxorubicin of resistant LoVo cells. We conclude that in LoVo cells drug resistance is associated with alteration of magnesium homeostasis through modulation of TRPM7. Our data suggest that TRPM7 expression may be an additional undisclosed player in chemoresistance.

Cancer exacts a very high toll as one of the leading causes of death all over the world. A complex hurdle in cancer treatment is the development of resistance to anticancer drugs, which is attributable to the expression of specific transporters that protect the neoplastic cells from harmful molecules^1^. However, it is increasingly evident that many other corollary mechanisms contribute to render cancer cells resistant to anti-neoplastic drugs. Among others, metals, which are essential in most cellular process, seem to be implicated. In particular, intracellular calcium (Ca) concentration is higher and its distribution within the cell is different in sensitive vs resistant cells^2^. Relatively little is known about magnesium (Mg), which is essential for life as a cofactor for ATP and hundreds enzymes, and also plays a part in intracellular signaling^3^.

Intracellular Mg is higher in cisplatin resistant than in sensitive ovarian carcinoma cells^4^. In drug resistant gastric cancer cells, the mitochondrial Mg transporter Mrs2 is upregulated and this inhibits doxorubicin (DXR)-induced apoptosis^5^.

In general, intracellular Mg homeostasis is maintained by the ubiquitously expressed ion channel transient receptor potential melastatin (TRPM)7, which shows the functional duality of being an ion channel and a kinase^6^. TRPM7 shares a very high homology with TRPM6, which is particularly abundant in the epithelium of the colon and the kidney and mainly involved in regulating body Mg balance^7^,^8^. TRPM6 and 7 are not functionally redundant^8^,^9^. Accordingly, they do not complement for each other’s deficiency in TRPM7^-/-^ B lymphocytes^10^, in TRPM6^-/-^ and TRPM7^-/-^ mice^11^,^12^. It is noteworthy that TRPM6 and 7 kinases have different substrate specificity. In particular, while both TRPM6 and 7 autophosphorylate their threonine residues, TRPM6 phosphorylates TRPM7, but not the opposite^9^. TRPM7 stands as the most intriguing of the two chanzymes as it is associated with a wide variety of biological functions^13^. It is noteworthy that the kinase activity is not essential for channel gating and *in vitro* annexin A1 and myosin IIA heavy chain have been shown to be substrates for the TRPM7 kinase^8^. In addition, a recent study has demonstrated that TRPM7’s kinase domain is proteolitically released from the channel and kinase cleaved fragments translocate to the nucleus and bind multiple components of chromatin-remodeling complexes, thus modulating the phosphorylation of specific histones^14^.

TRPM7 over-expression has been described in various human cancer cells including pancreatic, breast, ovarian, and head and neck carcinoma cells^15^,^16^,^17^,^18^, but no data are available about the expression of TRPM7 in drug sensitive or resistant tumor cells.

In turn, little is known about TRPM6 and cancer. A Boolean-based system biology approach has predicted TRPM6 as a potential drug candidate to prevent tumor growth^19^.

We performed our studies on colon carcinoma LoVo cells which are DXR-sensitive or resistant (LoVo-S and LoVo-R, respectively). Differences in energy metabolisms between LoVo-S and LoVo-R have been described^20^. In particular, the enhancement of energy-yielding pathways in LoVo-R correlates with higher levels of ATP than in LoVo-S^20^. Accordingly, a proteomic analysis has shown the differential expression of proteins involved in energy and detoxification pathways, cell survival, and chaperone function^21^. Recently, using a novel quantitative chemical imaging approach, we have shown the different Mg compartmentalization in LoVo-R compared to LoVo-S. In LoVo-R Mg was particularly abundant in the nucleus, while in LoVo-S Mg was mainly in the perinuclear region^22^.

We here measure total and free intracellular Mg, Mg fluxes, and the expression of TRPM6 and 7 in LoVo-R and -S. We also investigate the role of TRPM7 in modulating the sensitivity of LoVo cells to DXR and demonstrate that in this model of colon carcinoma cells drug resistance is associated with alteration of Mg homeostasis through modulation of TRPM7.

## Results

### Mg homeostasis in LoVo cells

**Figure 1:**
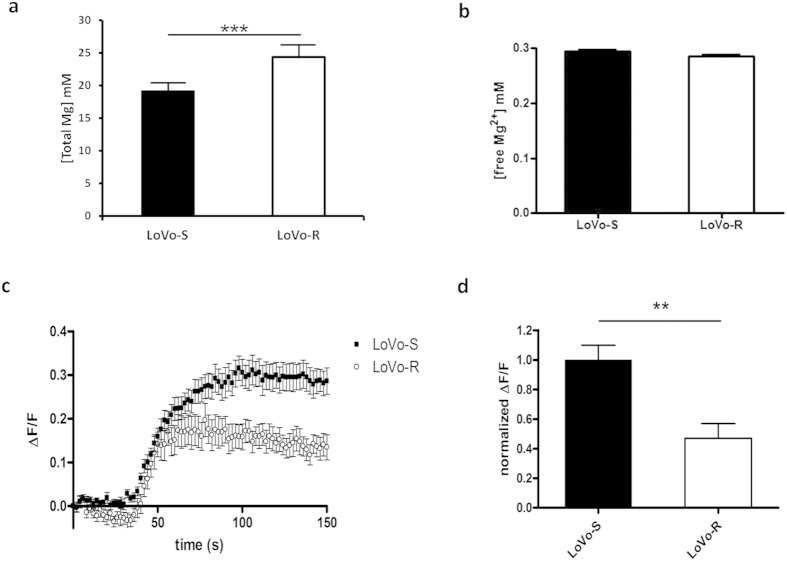
Mg levels and influx in LoVo-S and LoVo-R. (**a**) Total Mg was measured using the fluorescent chemosensor DCHQ5 as described. (**b**) Free Mg was measured using Mag-Fura-2-AM as described. (**c**) Time course of magnesium influx in Mag–Fluo-4-loaded cells was performed by live confocal imaging and single-cell fluorescence was evaluated by image analysis. The mean fluorescence (ΔF/F) of 10 cells ± standard deviation from a representative experiment is reported. (**d**) Fluorescence increment in LoVo-S and LoVo-R cells was measured at t = 150 s and normalized to LoVo-S cell values.

Initially we measured total and free Mg in LoVo cells. Total Mg was higher in LoVo-R than in LoVo-S (Fig. 1a), while free Mg was not significantly altered (Fig. 1b).

We then investigated Mg influx capacity in Mag-Fluo-4-loaded LoVo-S and LoVo-R by live confocal imaging^23^. As shown in Fig. 1c, LoVo-S intracellular fluorescence displayed a rapid and consistent increase reaching a plateau within 2 minutes. At plateau LoVo-R showed an intracellular fluorescence increase which accounts for 50% of the increase observed in LoVo-S (Fig. 1d). Mg efflux did not display any significant difference in LoVo-S and R in our experimental conditions (Supplementary Fig. S1).

**Figure 2:**
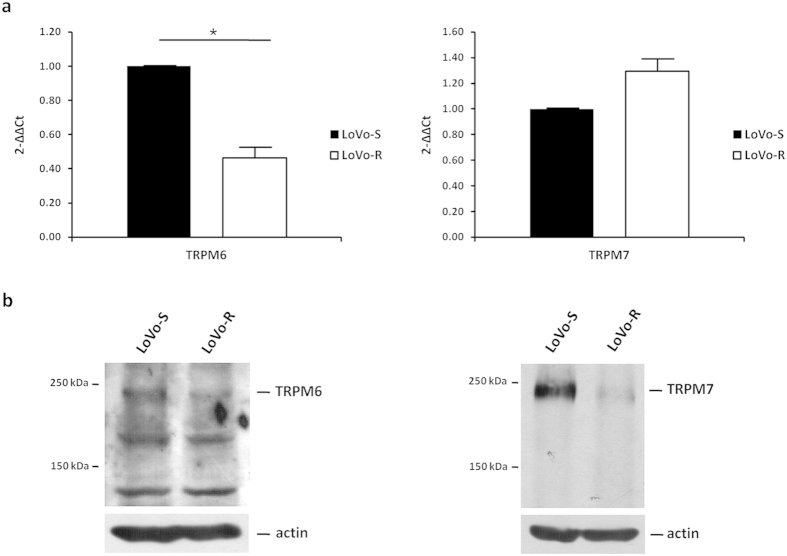
TRPM6 and 7 expression in LoVo-S and LoVo-R. (**a**) Real-Time PCR was performed on RNA extracted from LoVo cells using primers designed on *TRPM6* and *7* sequence. (**b**) LoVo cells were lysed and utilized for Western blot using antibodies against TRPM6 and 7. Actin was used as a control of loading. A representative blot is shown.

To get insights into these puzzling results, we evaluated the expression of TRPM6 and 7, the first molecularly defined components of the mammalian Mg transport machinery. LoVo-R expressed significantly lower amounts of *TRPM6* RNA than LoVo-S, while the levels of *TRPM7* RNA were comparable in the two cell types as detected by real time-PCR (Fig. 2a). By western blot, however, the total amounts of both TRPM6 and 7 were markedly reduced in LoVo-R in respect to LoVo-S (Fig. 2b-Supplementary Fig. S2).

### The contribution of calpains to Mg homeostasis in LoVo cells

**Figure 3:**
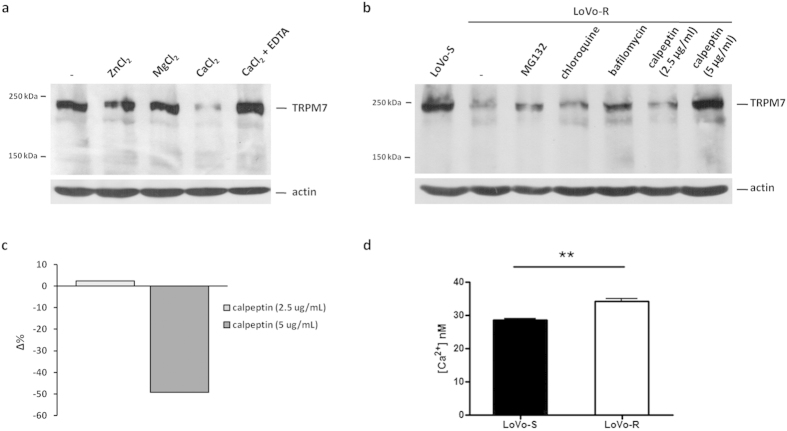
Ca-dependent TRPM7 degradation *in vitro* and *in vivo*, and effect on total Mg level. (**a**) LoVo-S cell extracts were incubated in a buffer containing 3 mM of CaCl_2_, MgCl_2_ or ZnCl_2_ for 30 min at room temperature. One sample was incubated with 3 mM of CaCl_2_ in the presence of EDTA (10 mM). The samples were immunoblotted with an antibody against TRPM7. (**b**) LoVo-R were treated with MG132 (5 μM), bafilomycin (100 nM), chloroquine (100 μM) or calpeptin (2.5 or 5.0 μg/ml) for 16 h at 37 °C. Western blot was performed on cell lysates with anti-TRPM7 antibodies. Actin was used to show that equal amounts of proteins were loaded per lane. (**c**) After 16 h exposure to calpeptin (2.5 or 5.0 μg/ml), total Mg was measured using the fluorescent chemosensor DCHQ5 as described. Data are shown as the change in Δ% of intracellular total Mg content in calpeptin treated LoVo-R cells referred to untreated LoVo-R cells. (**d**) Intracellular free Ca was measured using Fura-2-AM as described.

We investigated whether the reduced amounts of TRPM7 in LoVo-R could be due to degradation by intracellular proteases. Initially, we performed some studies *in vitro* on LoVo-S cell lysates to evaluate whether different cations, which are cofactors for several proteases, influenced the stability of TRPM7. Cell lysates were incubated in the presence of 3 mM of CaCl_2_, MgCl_2_ or ZnCl_2_ for 30 min at room temperature. While the addition of ZnCl_2_ and MgCl_2_ had no effects on TRPM7 levels, the addition of CaCl_2_ caused a marked reduction of the amounts of the protein. The Ca chelator EDTA prevented the degradation of TRPM7 (Fig. 3a-Supplementary Fig. S3A). These data demonstrate that Ca triggers TRPM7 degradation. We then evaluated TRPM7 levels in LoVo-R cultured in the presence of the proteasome inhibitor MG132 (5 μM), the calpain inhibitor calpeptin (2.5 and 5 μg/ml), chloroquine (100 μM) and bafilomycin (100 nM), which inhibit lysosomal activity. We found that, while bafilomycin, chloroquine and MG132 had little effect (less than 1.3 fold induction), 5 μg/ml calpeptin markedly increased TRPM7 in LoVo-R to levels comparable to those detected in LoVo-S (Fig. 3b- Supplementary Fig. S3B). To this purpose, it is noteworthy that calpeptin did not affect cell viability at the concentrations used as detected by MTT assay (Supplementary Fig. S3C).

The increment of TRPM7 by calpeptin was associated with a significant dose-dependent reduction of total intracellular Mg (Fig. 3c), thus indicating that in these cells an inverse correlation exists between total intracellular Mg and the levels of TRPM7.

Because Ca i) is known to activate calpains^24^ and ii) is higher in resistant than in sensitive cells^2^, we measured intracellular free Ca and found that it is significantly higher in LoVo-R than in LoVo-S (Fig. 3d).

### The contribution of TRPM7 to Mg homeostasis in LoVo cells

**Figure 4:**
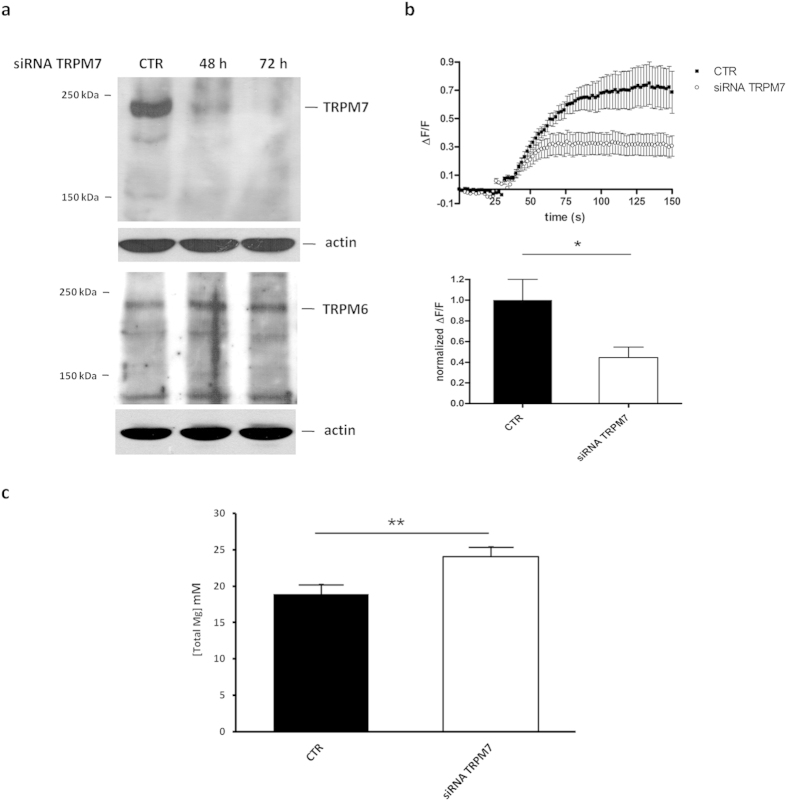
*TRPM7* silencing, Mg influx and concentration in LoVo-S. (**a**) LoVo-S were transfected with a siRNA against *TRPM7*. Cell extracts obtained 48 and 72 hours after transfection were run on two separate gels under the same experimental conditions. Western blot was performed using antibodies against TRPM6 and 7. Actin was used as a control of loading. (**b**) Time course of Mg influx in control (CTR) and *TRPM7*-silenced LoVo-S. Cells were transfected with a *TRPM7*-specific siRNA and analysed by live confocal imaging after 72 h (upper panel). Fluorescence increment in control and *TRPM7*-silenced LoVo-S was measured at t = 150s and normalized to control values (lower panel). (**c**) Total intracellular Mg was measured in LoVo-S after silencing *TRPM7* using the fluorescent chemosensor DCHQ5.

We focused our studies on the role of TRPM7 in regulating Mg homeostasis in our experimental model by transiently transfecting LoVo-S with specific siRNAs or with a non-silencing siRNA sequence as a control. The silencing of *TRPM7* was confirmed by western blot. A significant reduction in the total amounts of the channel was observed 48 and 72 hours after transfection (Fig. 4a- Supplementary Fig. S4). It is noteworthy that silencing *TRPM7* does not impact on the levels of TRPM6 (Fig. 4a- Supplementary Fig. S4).

We then measured Mg influx in Mag-Fluo-4-loaded LoVo-S after silencing *TRPM7* and found it significantly reduced, thus confirming that TRPM7 involvement in modulating Mg transport is fundamental in LoVo cells (Fig. 4b). However, total intracellular Mg was significantly increased in cells silencing TRPM7 (Fig. 4c). These results show that LoVo-S silencing *TRPM7* mimic LoVo-R since low levels of TRPM7 are associated with an increased intracellular concentration of total Mg.

### The contribution of TRPM7 to DXR resistance in LoVo cells

**Figure 5:**
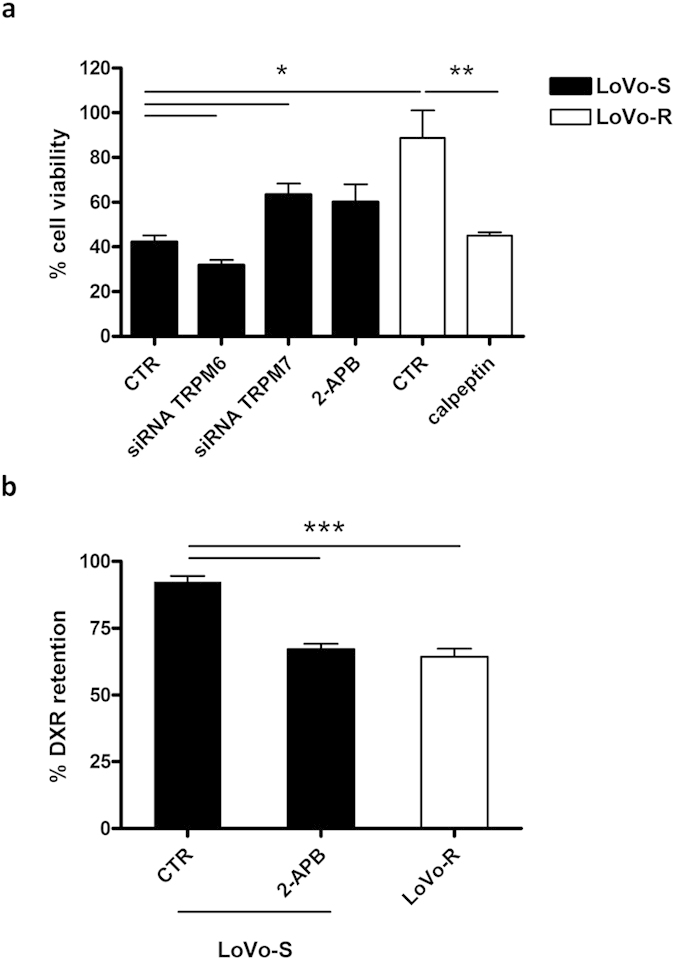
Modulation of *TRPM7* expression and/or activity affects sensitivity of LoVo cells to DXR. (**a**) Where indicated, LoVo-S were either silenced for *TRPM6* or *TRPM7* as detailed in the Materials and Methods section, or pretreated with the TRPM7 channel blocker 2-APB (50 μM), while LoVo-R were exposed to calpeptin (5 μg/ml). After 16 h the cells were challenged with DXR (1 μg/ml) for 48 h. Cell viability after DXR treatment was assessed by MTT assay. Percentage cell viability was calculated from the absorbance measured in DXR-treated *vs.* untreated cells for each condition. Data are mean ± SEM (n = 4). Statistical significance was assessed by Student’s t test referred to control LoVo-S (CTR, black bar) or control LoVo-R (CTR, white bar). (**b**) Cells were incubated with DXR for 2 h and were either immediately fixed (loading control) or allowed further 30 min in medium without DXR before fixing (efflux). Percentage DXR retention was quantified as the mean nuclear fluorescence measured in efflux conditions *vs.* loading control. Data are mean ± SEM (n = 80). Where indicated, LoVo-S cells were pretreated with 2-APB (50µM, 15 h). Statistical significance was assessed by Student’s t test referred to control LoVo-S (CTR).

To find a causal relationship between TRPM6/TRPM7 function and drug resistance in LoVo cells, LoVo-S were silenced for *TRPM6* and *TRPM7* or treated with TRPM7 channel blocker 2-aminoethoxydiphenyl borate (2-APB, 50µM). 15 hours later, the cells were challenged with DXR (1 μg/ml) and their viability was assessed by MTT assay. As shown in Fig. 5a, after DXR treatment about 42% of LoVo-S remained viable *vs.* 89% of LoVo-R, as expected. While silencing *TRPM6* (Supplementary Fig. S5A) reduced cell survival, silencing *TRPM7* increased cell survival (about 63% of viable LoVo-S after DXR treatment). 2-APB markedly increased cell survival without reaching the statistical significance. However, when we exposed LoVo-S to 2-APB for 15 h and then measured DXR retention, we found that LoVo-S behave like LoVo-R and retain lower amounts of DXR (Fig. 5b and Supplementary S5B).

We also treated LoVo-R with calpeptin and then challenged them with DXR. Calpeptin, which upregulates TRPM7 and decreases intracellular Mg, renders the cells more sensitive to the toxic effect of DXR (Fig. 5a).

Overall, these results suggest that interfering either with the expression or the activity of TRPM7 affects the sensitivity of LoVo cells to DXR.

## Discussion

High intracellular Mg selectively advantages neoplastic cells, since it reprograms metabolism, activates telomerase and inactivates p53 through the inhibition of the nuclear Ser/Thr phosphatase PPM1D^25^. A link between low extracellular magnesium and the risk of colon cancer has been shown^25^. Moreover, a single nucleotide polymorphism that substitutes TRPM7 threonine 1482 (T1482) to isoleucine (T1482I) increases the risk of development of colon cancer^25^,^26^. Interestingly, TRPM7 T1482 is a potential site of phosphorylation by TRPM6. It is therefore possible to hypothesize a different regulation of the TRPM7 T1482I channel by TRPM6, which might be involved in promoting the acquisition of a neoplastic phenotype. On these bases, TRPM6 and 7 are emerging as puzzling, potential players in colon cancer.

We here investigate the cellular handling of Mg in human colon carcinoma cells sensitive or resistant to DXR and its contribution to drug resistance.

By a novel quantitative chemical imaging approach that combines synchrotron radiation microscopy with atomic force microscopy, we have recently shown a higher concentration of total intracellular Mg in LoVo-R than LoVo-S, and a different pattern in the spatial distribution of molar Mg concentration^22^. In particular, LoVo-R displayed the highest values of molar Mg concentration within the nucleus, while LoVo-S showed the highest values in the peri-nuclear region. This feature was not evident for all the other elements evaluated in this study, which suggests that Mg compartmentalization could be a significant trait of the drug resistant cells^22^. We here report quantitative data showing the increase of total intracellular Mg in LoVo-R vs LoVo-S, thus providing additional evidence on the different content of total intracellular Mg in the two cell types. It is noteworthy that total Mg content was higher also in cisplatin-resistant human ovarian carcinoma cells when compared to parental sensitive cells and this seems to be responsible for the impairment of apoptosis in resistant cells^4^. In LoVo-S and -R, we found no significant differences in free Mg. This result is in agreement with previous studies showing that intracellular cytosolic free Mg is relatively unchanged during cellular massive translocation of total Mg^27^,^28^. Thanks to those pioneering studies the notion that free intracellular Mg and total intracellular Mg behave differently and are differently regulated is a consolidated concept and finds here additional support.

Significant increases in free Mg are observed only in dramatic metabolic perturbations, i.e. after ischemia, while after physiologic signaling cytosolic free Mg remains constant. Not only cytosolic free Mg was the same in LoVo-R and -S, but also we could not detect significant differences in Mg efflux between LoVo-R and -S.

We hypothesize that the higher concentration of total intracellular Mg in spite of its reduced influx might be explained by the different metabolic profile of LoVo-R vs LoVo-S that accounts for different concentrations of buffering components^20^.

It is noteworthy that the levels of TRPM7 are inversely related to the amounts of total intracellular Mg. Briefly, LoVo-R, which have higher intracellular Mg than LoVo-S, downregulate TRPM7. Conversely, LoVo-S silencing TRPM7 have more intracellular Mg than controls. Interestingly, the same results were obtained in human endothelial cells derived from the umbilical vein. Indeed, high extracellular Mg, which increases total intracellular Mg^29^, downregulates TRPM7 through the activation of calpains, intracellular Ca-dependent cysteine proteases which are involved in a large number of physiological and pathological events^24^. Accordingly, TRPM7 was markedly reduced in resistant cells because of the activation of calpains. Since calpains are Ca-dependent, it should be recalled that free Ca is higher in LoVo-R than in LoVo-S, as previously demonstrated in different cell types resistant to drugs^2^. In Lovo-R calpeptin, a specific calpain inhibitor which prevents the binding and subsequent proteolysis of calpain substrates^30^, augments TRPM7 to levels comparable to those found in LoVo-S and, in agreement with the results discussed above, decrease the total amount of intracellular Mg. Therefore calpains are emerging as important modulators of Mg homeostasis. Interestingly, TRPM7 is a potent regulator of m-calpain and co-localizes with the enzyme at peripheral adhesion complexes in HEK293 cells^31^,^32^. On these bases, we propose the existence of an interplay between calpains and TRPM7 where TRPM7 is both a regulator and a target of these enzymes.

Also TRPM6 is downregulated in LoVo-R. The very low levels of TRPM6 might be explained in the light of a transcriptional regulation, because both mRNA and protein are lower in drug resistant than in sensitive cells. Since TRPM6 is needed for Mg absorption by epithelial cells and contributes to systemic Mg homeostasis^7^, we hypothesize that resistant cells are less differentiated than sensitive cells, but more studies are needed to clarify this issue.

TRPM6 seems to function when assembled with TRPM7^33^. Indeed, even though the stoichiometry remains to be elucidated, TRPM6 and 7 both participate to the heteromeric channel pore^34^ and recent data indicate that the heteromeric TRPM6/7 channel is more efficient in regulating divalent ion influx than homomeric channels constituted by TRPM6 or 7 alone^35^. Recently, in HEK-293 cells, TRPM6 has also been shown to adjust the cell distribution of TRPM7 through its kinase activity^34^. Since i) low amounts of TRPM7 decrease Mg influx in LoVo-R and ii) silencing TRPM7 in LoVo-S reduces Mg entry, our results suggest that TRPM7 is a crucial channel for Mg entry in LoVo cells.

More importantly, we provide evidence that TRPM7 is involved in modulating drug resistance in LoVo cells. Indeed, downregulating *TRPM7*, but not *TRPM6*, expression by siRNA enhances cell viability of LoVo-S exposed to DXR. On the other hand, calpeptin, which upregulates TRPM7, renders LoVo-R more sensitive to the toxic effect of DXR. We therefore hypothesize that a link exists between low amounts of TRPM7, high intracellular magnesium and drug resistance. Since TRPM7 is also a kinase^8^,^9^,^10^,^11^,^12^,^13^,^14^, its inhibition might alter the phosphorylation of downstream targets, eventually contributing to the regulation of drug resistance.

We conclude that Mg homeostasis and the expression of two Mg channels, TRPM6 and 7, are altered in drug resistant colon carcinoma cells. In particular, *TRPM6* may represent a key player in this model, as *TRPM7* silencing appears to shift the phenotype of sensitive LoVo cells to one more similar to resistant cells. These findings reinforce previous reports underscoring the existence of a Mg-regulating mechanism in drug resistance^4^,^36^, and suggest that TRPM7 expression and/or activity may constitute novel targets for therapeutic intervention and circumvention of chemoresistance.

## Methods

### Cell culture

Colon cancer LoVo cells sensitive (LoVo-S) or resistant to DXR (LoVo-R) (kindly donated by Dr. P. Perego, Istituto Nazionale Tumori, Milano) were cultured in DMEM containing 10% fetal bovine serum and 2 mM glutamine at 37 °C and 5% CO_2_.

To obtain a transient downregulation of *TRPM6* and *7*, we utilized the stealth siRNAs developed by Qiagen^37^. siRNAs were transfected into 2 × 10^4^/cm^2^ cells using HiPerFect Transfection Reagent (Qiagen). Non-silencing, scrambled sequences were used as controls (CTR).

To evaluate sensitivity to DXR, cell viability was assessed by MTT assay. Briefly, cells were seeded in 96 well/plates. Where indicated, 16 h prior to DXR treatment, LoVo-S were silenced for *TRPM7* or *TRPM6*, or exposed to 2-aminoethoxydiphenyl borate (2-APB, 50 μM), while LoVo-R were treated with calpeptin (5 μg/ml). After 48 h of DXR treatment (1 μg/ml), culture medium was replaced with medium containing 3-(4,5-dimethylthiazol-2-yl)-2,5 diphenyltetrazolium bromide (MTT, 0.5 mg/ml) (Sigma, Oakville, Ontario, Canada). At the end of the experiment, media were removed and formazan crystals generated by cellular reductase activity were dissolved in DMSO:ethanol (1:1) and quantified by absorbance readings at 575 nm. The experiment was repeated three times in quadruplicates. Percentage cell viability was calculated from the absorbance measured in DXR-treated *vs.* untreated cells for each experimental condition.

To assess DXR retention, cells were loaded with 10 μM DXR for 2 h and fixed either immediately (loading control) or 30 min after washing (efflux). Nuclear fluorescence was visualized by confocal microscopy and quantified as the mean nuclear fluorescence measured in efflux conditions *vs.* loading control.

### Real-Time PCR

Total RNA was extracted by the PureLink RNA Mini kit (Ambion). Single-stranded cDNA was synthesized from 0.2 μg RNA in a 40 μl final volume using High Capacity cDNA Reverse Transcription Kit, with RNase inhibitor (Applied Biosystems) according to the manufacturer’s instructions. Real-time PCR was performed in triplicate on the 7500 FAST Real Time PCR System instrument using TaqMan Gene Expression Assays (Life Technologies, Monza, Italy): Hs00918928_g1 (*TRPM7*) and Hs01019353_m1 (*TRPM6*). The housekeeping gene *GAPDH* (Hs99999905_m1) was used as an internal reference gene. Relative changes in gene expression (defined as FC) were analyzed by the 2^-ΔΔ*Ct*^ method^38^.

### Western blot analysis

Cells were lysed in lysis buffer (50 mM TrisHCl pH 8.0, 150 mM NaCl, 1 mM EDTA, 1% NP-40). Protein concentration was determined using the Bradford protein assay (Bio-Rad). Cell extracts (100 μg/lane) were resolved by 8% SDS-PAGE, transferred to nitrocellulose sheets at 100 mA for 16 h, and probed with anti-TRPM7 (Bethyl), anti-TRPM6 (Osenses), and anti-actin (Sigma Aldrich) antibodies. Secondary antibodies were labelled with horseradish peroxidase (GE Healthcare). The SuperSignal chemiluminescence kit (Pierce) was used to detect immunoreactive proteins. All the results were reproduced at least three times and a representative Western blot is shown. Full length blots are shown in the Supplementary Information. Densitometric analysis was performed by the ImageJ software and TRPM7 or 6/actin ratio was calculated on three separate experiments (see supplementary).

### Mg influx measurements

Subconfluent cells on 35-mm microscopy dishes (μ-dish, Ibidi) were loaded with 3 μM Mag-Fluo-4-AM (Invitrogen), and imaged in a Na, Ca and Mg-free buffer at a confocal laser scanning microscope, as previously described^39^. Cytosolic fluorescence signals were recorded as time series of 5 min at a sampling frequency of 30 frames/min. The baseline was monitored for 30 sec, then MgSO_4_ was added dropwise at a final concentration of 20 mM. Changes in intracellular Mg levels at single cell level were estimated by the mean fluorescent increment ΔF/F^38^. Image analysis was performed by Leica Confocal Software and 10 representative cells were examined in each microscopic field.

### Quantification of total cell Mg

Total Mg content was assessed on sonicated cells by using the fluorescent chemosensor DCHQ5^40^. Briefly, DCHQ5 was dissolved to a final concentration of 15 μM in a mixture which contains 10% of Phosphate Buffer Saline (PBS) in a solution 1:1 of MeOH:MOPS 2 mM (pH 7.4). To perform standard curve, different amounts of MgSO_4_ were added and the fluorescence intensities were acquired at 510 nm. Mg concentrations of the samples were obtained by the interpolation of their fluorescence with the standard curve and were referred to the actual cell volume measured as described in the next paragraph.

### Cell volume assessment

LoVo-S and LoVo-R were trypsinized and resuspended in PBS. Cells volume was calculated assessing the diameter cell profile by using a Beckman Coulter counter (Beckman Coulter). The cells volume is given in terms of equivalent spherical diameter. The analysis were carried out in triplicate.

### Quantification of free Mg and Ca

Cells were trypsinized, washed and resuspended at 2 × 10^6^/mL in loading buffer (LB) containing 120 mM NaCl, 20 mM HEPES, 4.7 mM KCl, 1.2 mM KH_2_PO_4_, 10 mM glucose, 1.2 mM CaCl_2_, and 1.2 mM MgSO_4_. Cells were loaded in LB containing 3 μM Mag-Fura-2-AM or Fura-2-AM (for Mg^2+^ and Ca^2+^ measurements, respectively) for 30 min at 37 °C, then washed twice and maintained further 30 min in LB to allow for complete de-esterification of AM esters. Analysis was performed at a FluoroMax-4 spectrofluorometer (Horiba Jobin Yvon) at 37 °C. Fura dyes were excited with dual excitation wavelengths at 330 and 369 nm and emission was monitored at 511 nm. Ratio calibration was performed by the in-built software HJY Multigroup, using R_min_ and R_max_ values obtained by sequential addition of 25 mM MgSO_4_, 0.05% Triton and 50 mM EDTA for Mag-Fura-2, or 10 mM CaCl_2_, 0.05% Triton and 10 mM EGTA for Fura-2. Dissociation constants of 1.5 mM^41^ or 224 nM^42^ were used for Mag-Fura-2-AM or Fura-2-AM, respectively.

### Statistical analysis

Statistical significance was determined using Student’s t test and set as following: *P < 0.05, **P < 0.01, ***P < 0.001.

## Additional Information

**How to cite this article**: Castiglioni, S. *et al.* Magnesium homeostasis in colon carcinoma LoVo cells sensitive or resistant to doxorubicin. *Sci. Rep.* **5**, 16538; doi: 10.1038/srep16538 (2015).

## Supplementary Material

Supplementary Information
