# Supplementary material for: Magnesium homeostasis in colon carcinoma LoVo cells sensitive or resistant to doxorubicin
Source: Sci Rep. 2015 Nov 13;5:16538. doi: 10.1038/srep16538 (PMC4643312; doi:10.1038/srep16538)

**Magnesium homeostasis in colon carcinoma LoVo cells sensitive or resistant to doxorubicin**

Sara Castiglioni, Alessandra Cazzaniga, Valentina Trapani, Concettina Cappadone, Giovanna Farruggia, Lucia Merolle, Federica I. Wolf, Stefano Iotti and Jeanette AM Maier

**Supplementary Figures**

**Figure S1**

**Mg^2+^ efflux capacity of LoVo cells**. Mag-Fluo-4 loaded cells were first loaded with 5 mM Mg^2+^ and then placed in a Mg^2+^-free buffer. Fluorescence signal was collected by confocal live imaging and single-cell fluorescence was evaluated by image analysis. The mean fluorescence (ΔF/F) of 10 cells ± SEM from a representative experiment is reported (n=3).

**
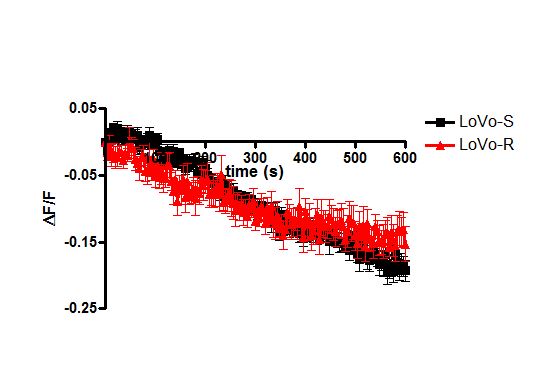
**

**Figure S2**

**Full length blot**

**
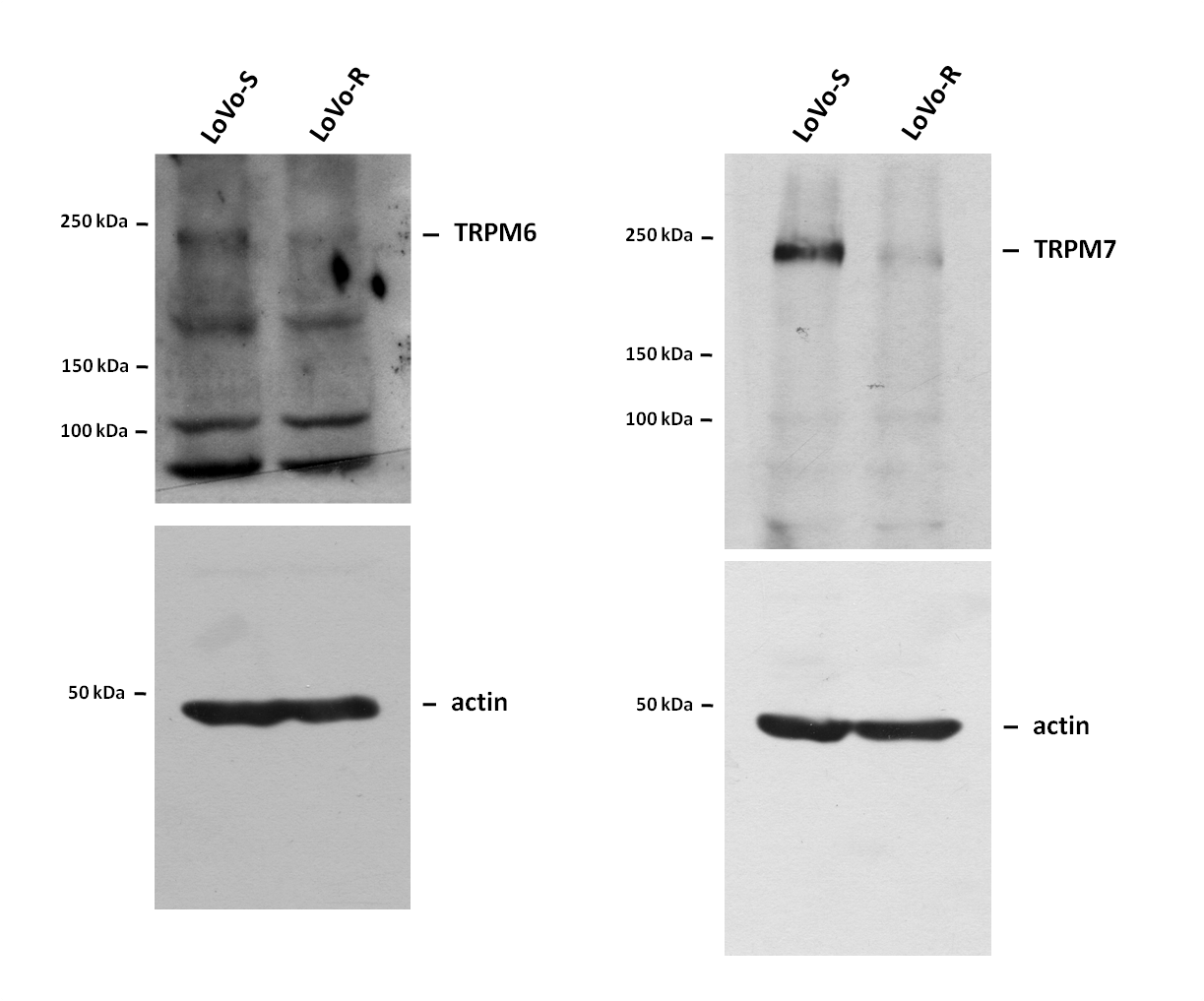
**

**TRPM6 and 7 expression in LoVo cells.** Protein levels were assessed by western blot.

Densitometric analysis was performed by the ImageJ software and TRPM7 or 6/actin ratio was calculated on three separate experiments. *P<0.05, **P<0.01.


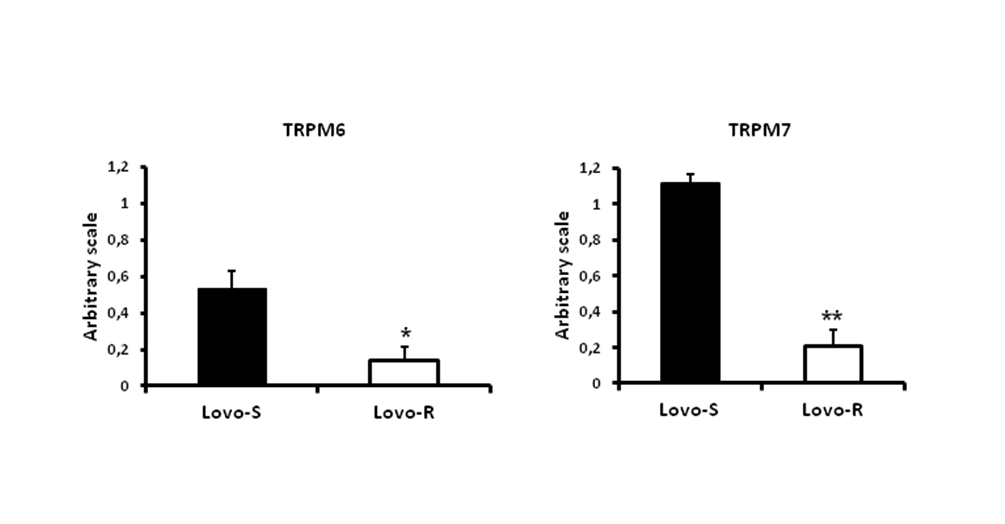


**Figure S3**

**Ca-dependent TRPM7 degradation *in vitro*. (A)** LoVo-S cell extracts were incubated in a buffer containing 3 mM of CaCl_2_, MgCl_2_ or ZnCl_2_ for 30 min at room temperature. One sample was incubated with 3 mM of CaCl_2_ in the presence of EDTA (10 mM). The samples were immunoblotted with an antibody against TRPM7. **(B)** LoVo-R were treated with MG132 (5 μM), bafilomycin (100 nM), chloroquine (100 µM) or calpeptin (2.5 or 5.0 µg/ml) for 30 min at 37 °C. Western blot was performed on cell lysates with anti-TRPM7 antibodies. Actin was used to show that equal amounts of proteins were loaded per lane.

**Full length blot**

**
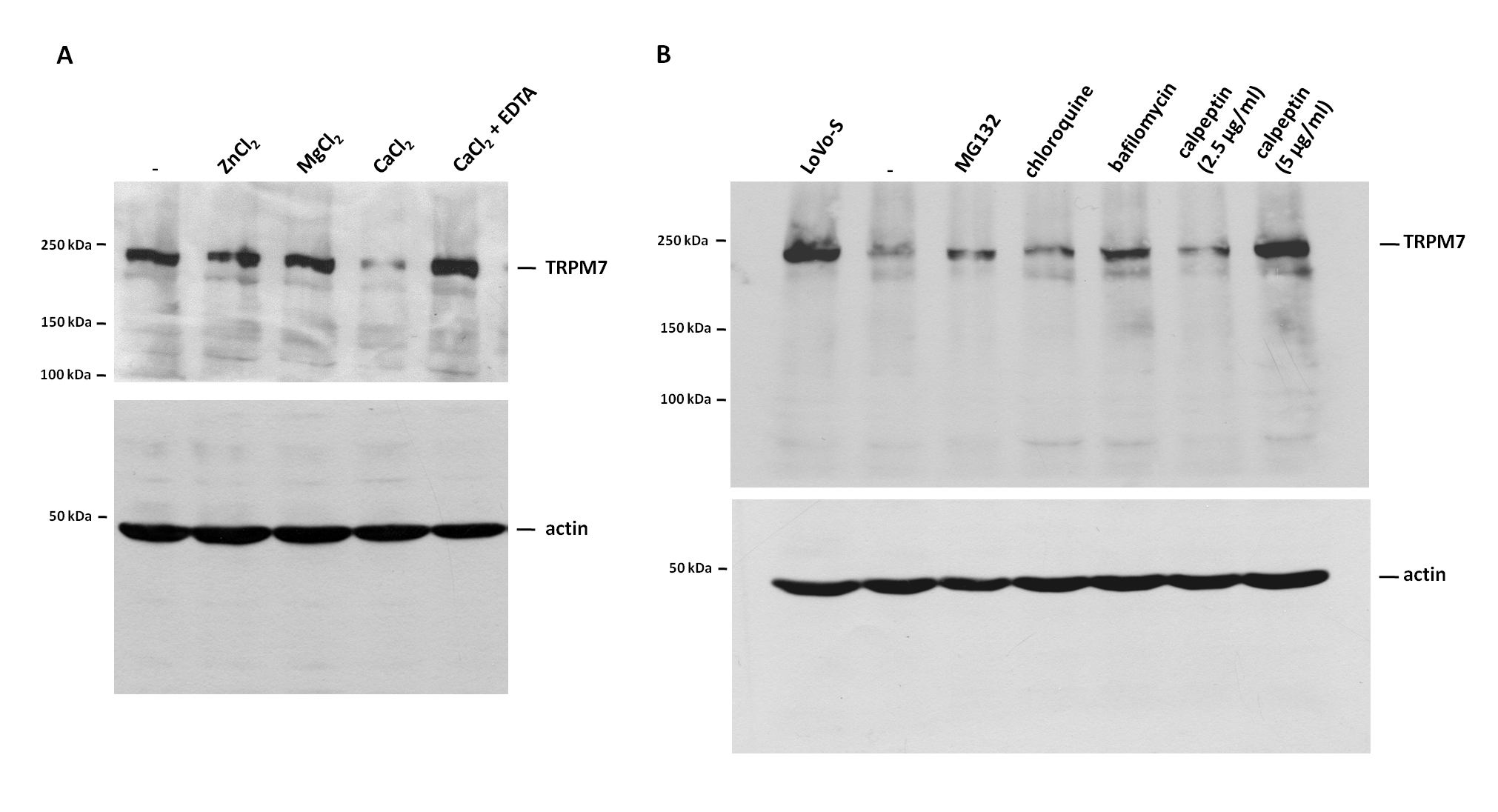
**

**Figure S3A**

Densitometric analysis was performed by the ImageJ software and TRPM7/actin ratio was calculated on three separate experiments. **P<0.01.
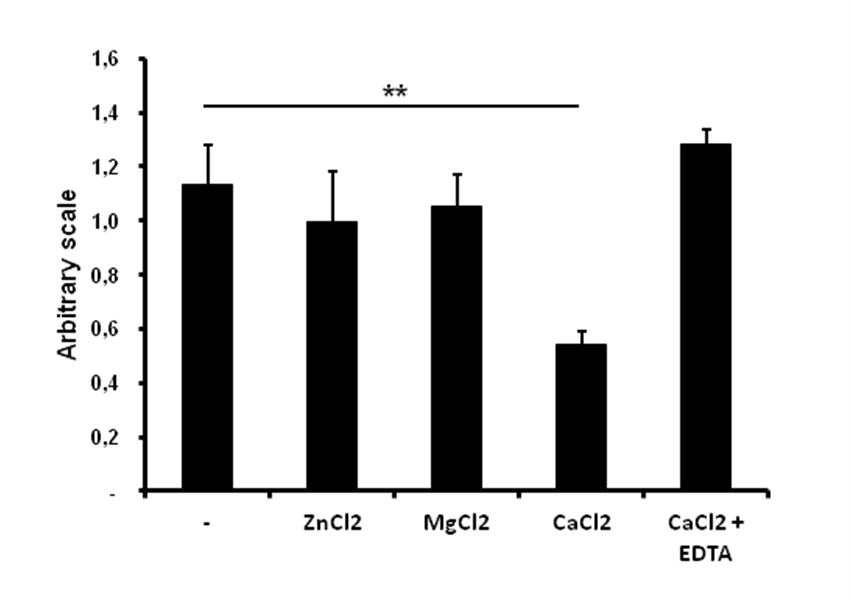


**Figure S3B**

Densitometric analysis was performed by the ImageJ software and TRPM7 /actin ratio was calculated on three separate experiments. **P<0.01


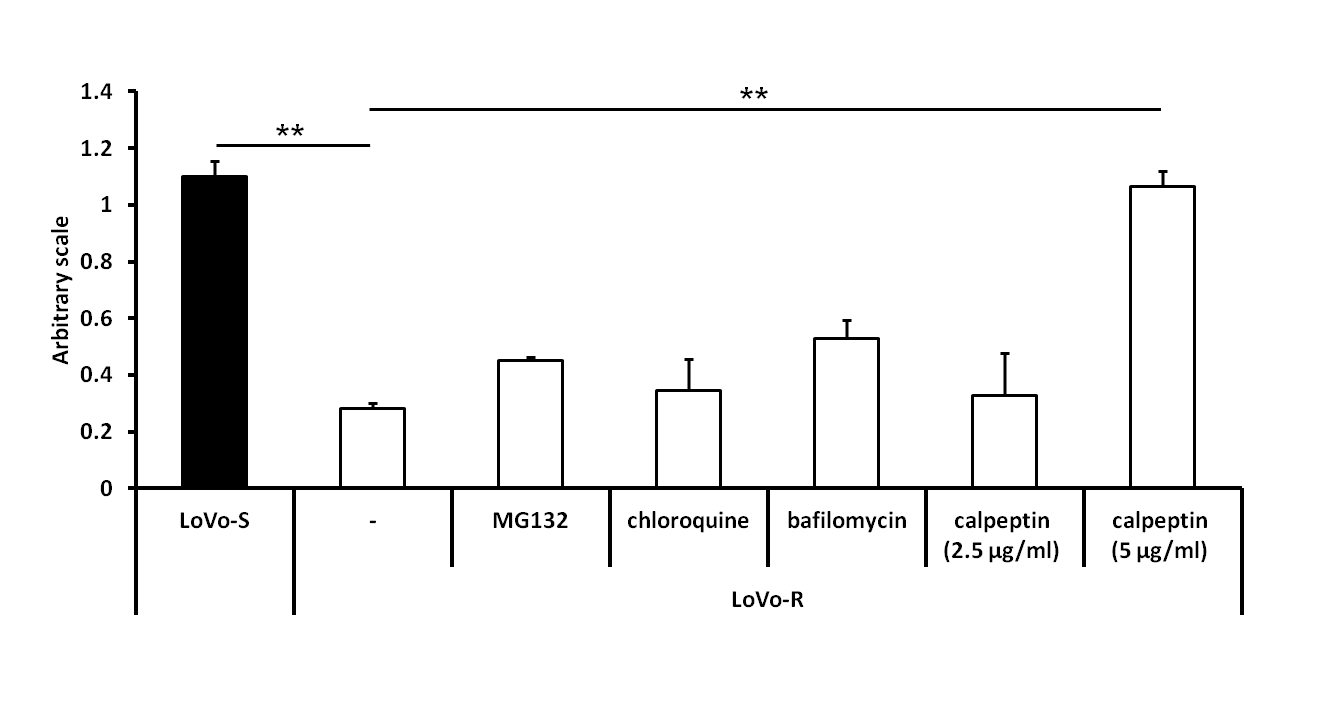


**Figure S3C**

**Cell viability after treatment with calpeptin**.

LoVo-R were treated with different concentrations of calpeptin and cell viability was assessed after 48 h by MTT assay. **P<0.01

**
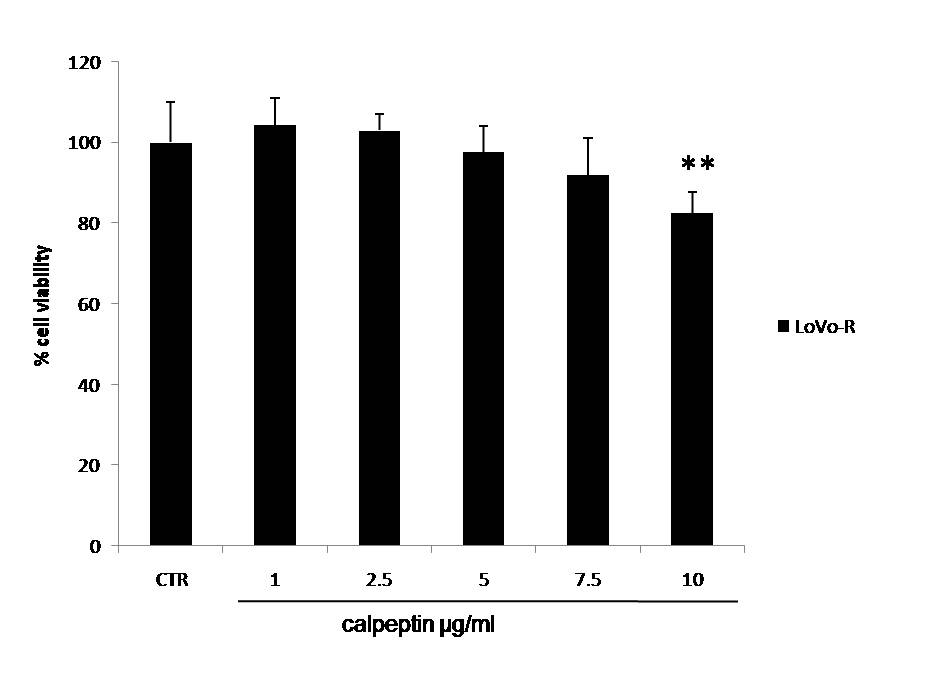
**

**Figure S4**

***TRPM7* silencing.**

LoVo-S were transfected with a siRNA against *TRPM7*. Western blot was performed on cell extracts 48 and 72 h after transfection using antibodies against TRPM6 and 7. Actin was used as a control of loading.

**Full length blots**

**
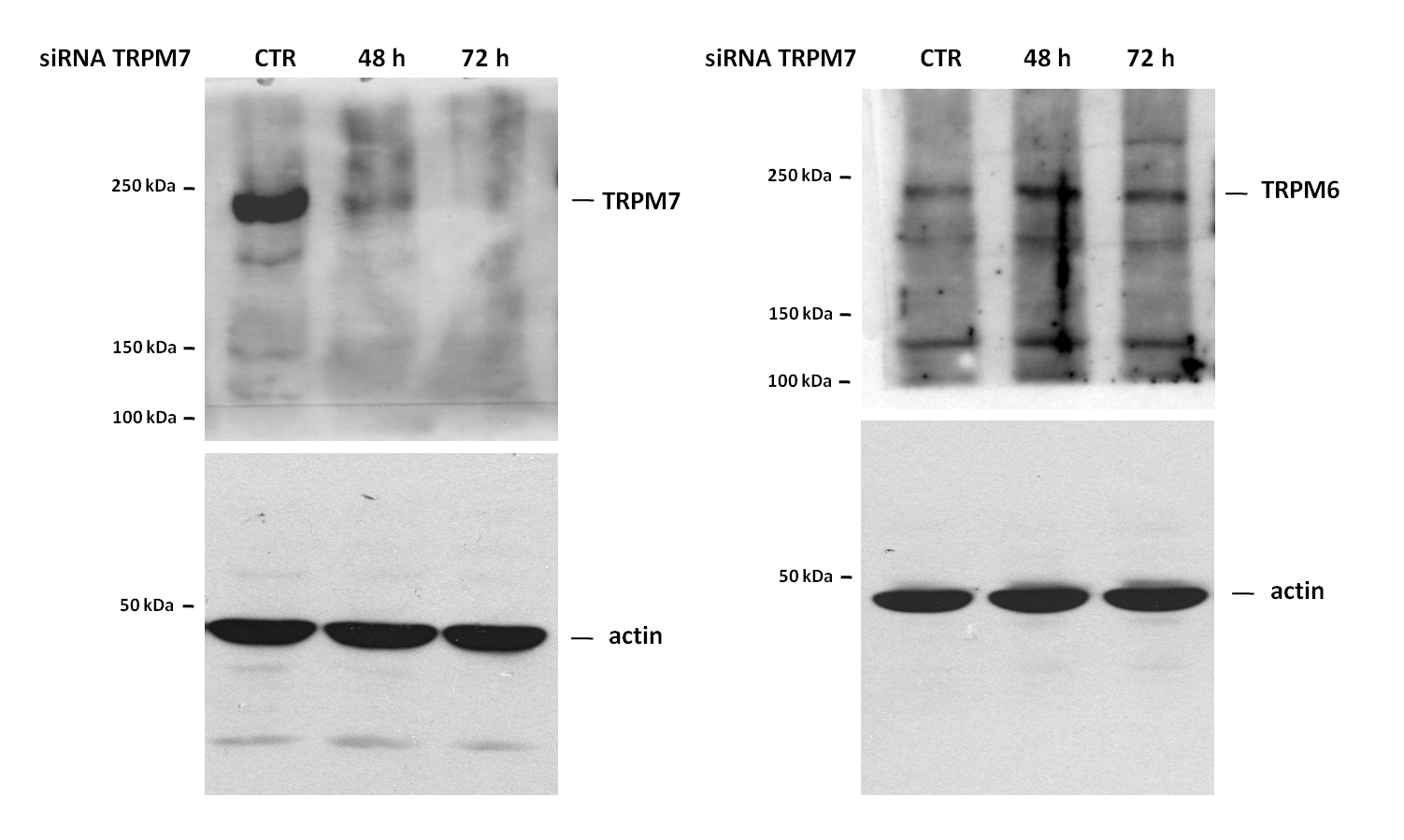
**

Densitometric analysis was performed by the ImageJ software and TRPM7 or 6/actin ratio was calculated on three separate experiments. **P<0.01, ***P<0.001.


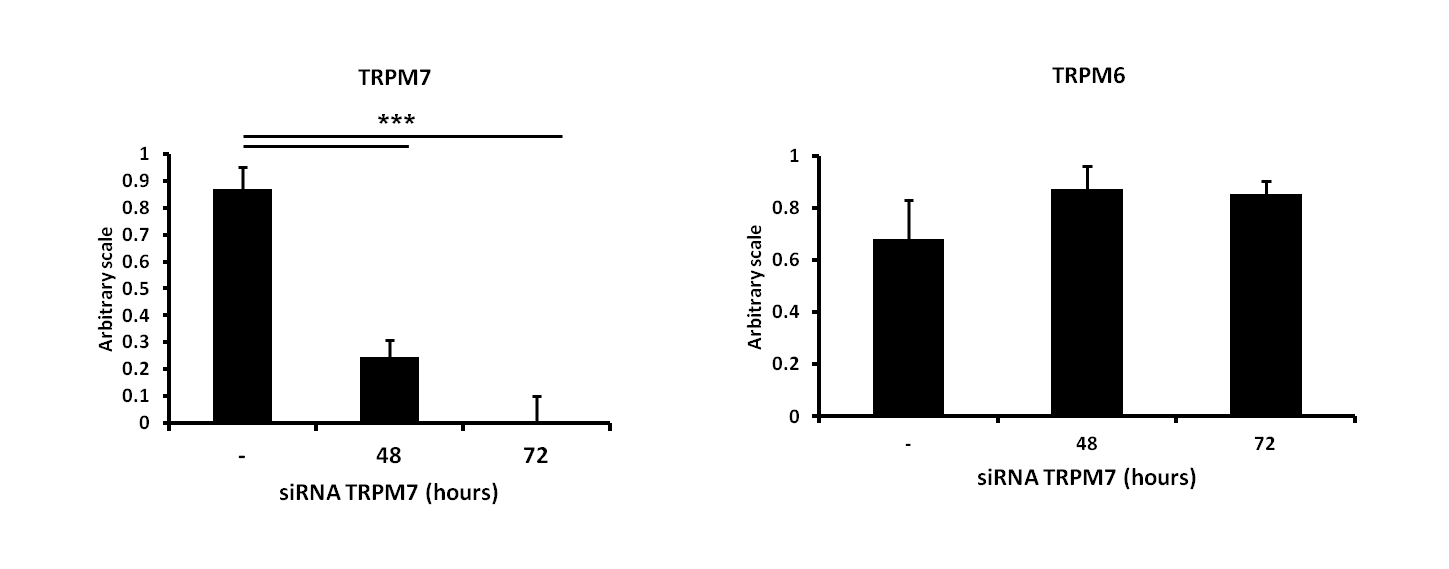


**Figure S5A**

**TRPM6 silencing.** To obtain a transient downregulation of *TRPM 6*, we utilized the stealth siRNAs developed by Qiagen [22]. siRNAs were transfected into 2×10^4^/cm^2^ cells using HiPerFect Transfection Reagent (Qiagen). Non-silencing, scrambled sequences were used as controls (CTR). Densitometric analysis was performed as above. ***P<0.001.


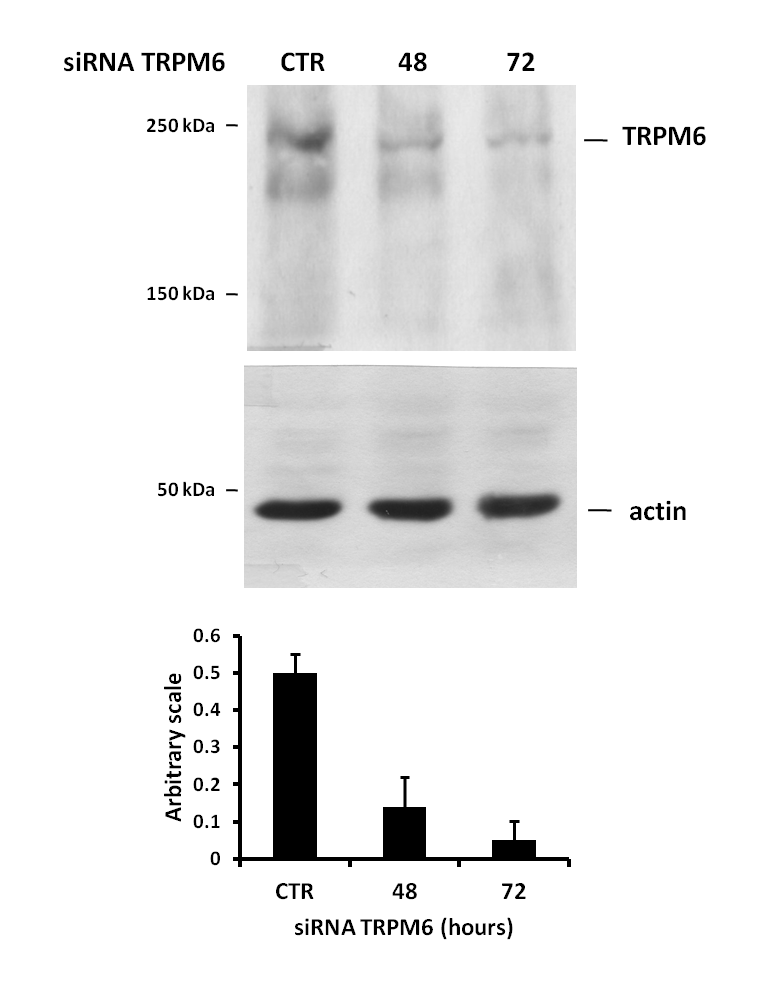


**Figure S5B.**

Inhibition of TRPM7 activity affects intracellular DXR retention of LoVo-S cells. Cells were incubated with DXR for 2h and were either immediately fixed (loading) or allowed further 30 min in medium without DXR before fixing (retention). Where indicated, LoVo-S cells were pretreated with 2-APB (50 μM, 15h). Nuclear fluorescence was visualized at a confocal microscope with a 40X magnification and identical acquisition parameters for all conditions.


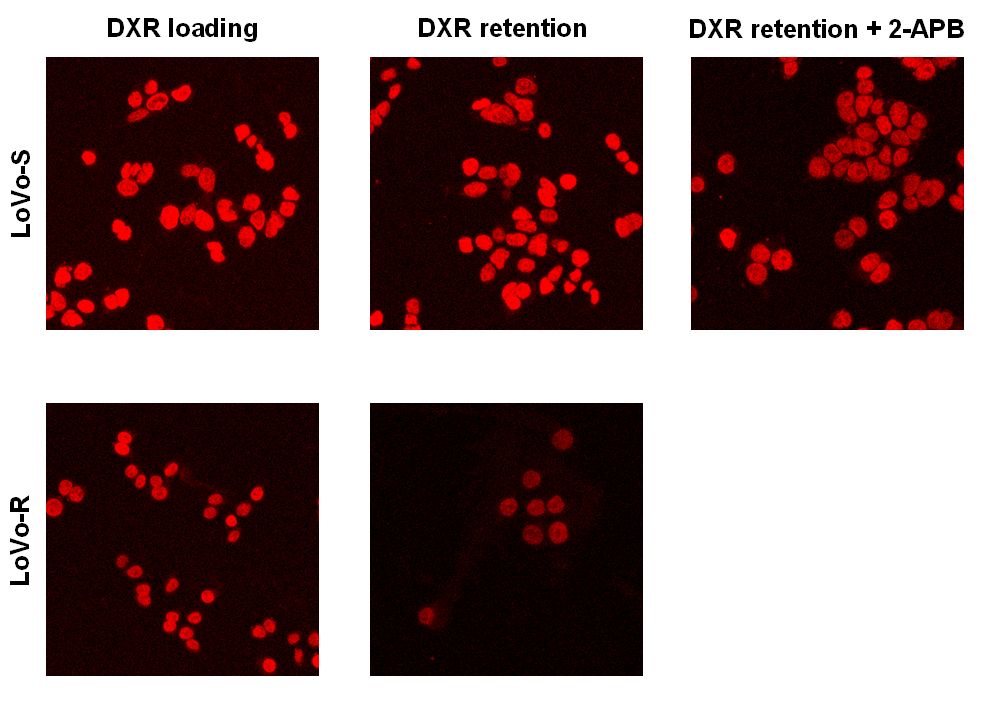

Supplement: Supplementary Information [file srep16538-s1.doc]
